# Optogenetic Manipulation of Olfactory Responses in Transgenic Zebrafish: A Neurobiological and Behavioral Study

**DOI:** 10.3390/ijms22137191

**Published:** 2021-07-03

**Authors:** Yun-Mi Jeong, Tae-Ik Choi, Kyu-Seok Hwang, Jeong-Soo Lee, Robert Gerlai, Cheol-Hee Kim

**Affiliations:** 1 Department of Biology, Chungnam National University, Daejeon 34134, Korea; angdoym@kribb.re.kr (Y.-M.J.); c860523@naver.com (T.-I.C.); kshwang@krict.re.kr (K.-S.H.); 2 Disease Target Structure Research Center, Korean Research Institute of Biosciences and Biotechnology, Daejeon 34141, Korea; jeongsoo@kribb.re.kr; 3 Department of Psychology, University of Toronto Mississauga, Mississauga, ON L5L 1C6, Canada

**Keywords:** zebrafish, olfaction, optogenetics, channelrhodopsin

## Abstract

Olfaction is an important neural system for survival and fundamental behaviors such as predator avoidance, food finding, memory formation, reproduction, and social communication. However, the neural circuits and pathways associated with the olfactory system in various behaviors are not fully understood. Recent advances in optogenetics, high-resolution in vivo imaging, and reconstructions of neuronal circuits have created new opportunities to understand such neural circuits. Here, we generated a transgenic zebrafish to manipulate olfactory signal optically, expressing the Channelrhodopsin (ChR2) under the control of the olfactory specific promoter, *omp*. We observed light-induced neuronal activity of olfactory system in the transgenic fish by examining *c-fos* expression, and a calcium indicator suggesting that blue light stimulation caused activation of olfactory neurons in a non-invasive manner. To examine whether the photo-activation of olfactory sensory neurons affect behavior of zebrafish larvae, we devised a behavioral choice paradigm and tested how zebrafish larvae choose between two conflicting sensory cues, an aversive odor or the naturally preferred phototaxis. We found that when the conflicting cues (the preferred light and aversive odor) were presented together simultaneously, zebrafish larvae swam away from the aversive odor. However, the transgenic fish with photo-activation were insensitive to the aversive odor and exhibited olfactory desensitization upon optical stimulation of ChR2. These results show that an aversive olfactory stimulus can override phototaxis, and that olfaction is important in decision making in zebrafish. This new transgenic model will be useful for the analysis of olfaction related behaviors and for the dissection of underlying neural circuits.

## 1. Introduction

Multimodal integration of various sensory information in the environment is important to generate appropriate behavioral responses. How the brain transforms sensory information into an observable behavioral output, i.e., how a decision is made to respond to a stimulus, is a major question in neuroscience. When two sensory inputs conflict, one sensory influence the other and this dominance may be beneficial in decision making of animals [1]. Auditory information dominates visual cue in certain circumstances [1], and olfactory stimuli often modulates visual perception [2]. However, when incompatible sensory inputs such as an attractive and an aversive cue were given concurrently, which cue would be dominance and the underlying mechanism remains unclear. Olfaction (i.e., sense of smell) is one of the most important senses affecting decision making for survival and fundamental behaviors of animals [3].

Zebrafish is an attractive vertebrate model to study sensory processing in the nervous system for numerous reasons, one of which is that this species possesses a complex behavioral repertoire and exhibits behavioral responses that can be induced reliably [3]. The olfactory organ of fish is well developed to perceive odorants in an aquatic environment [4]. Recognition of odors from spoiled food or predators can be important to elicit suitable avoidance behaviors for survival of animal [5]. Zebrafish larvae also respond to visual stimuli. For example, zebrafish larvae show phototaxis whereby the fish swim toward the light [6]. Furthermore, zebrafish larvae spend more time in brightly illuminated areas when placed in a light/dark choice task, a response that is a matter of controversy in adults [7,8,9,10,11,12]. Along these lines, zebrafish larvae also exhibit complex behavioral responses including spontaneous motor behavior as well as learning and memory [13,14].

Optogenetics is an emerging field that combines genetics and systemic stimulation with a specific wavelength of light to achieve precise temporal control over gain or loss-of-function within specific cells of living organisms [15,16,17]. For example, microbial opsin genes can be introduced to control action potential in specific neuronal populations within freely moving mammals or other intact animal model system. Nagel and colleagues employed channelrhodopsin-2 (ChR2), which is a light-gated ion channel, in excitable cells of the nematode to trigger specific behaviors by blue light (480 nm), and in zebrafish, photo-activation of ChR2 in the somatosensory neurons were reported to trigger escape behavior [18,19].

In the current study, we generated a transgenic zebrafish that enables us to control perception of olfactory stimuli. For this, we used optogenetic molecular tools such as ChR2 and Kaede, which is a photo-convertible fluorescent protein. We generated a transgenic fish to stimulate the olfactory sensory neurons with blue light using these optogenetic molecules, ChR2 and Kaede. We made a fusion protein of ChR2 and Kaede, and expressed this construct in zebrafish olfactory sensory neurons under the control of *omp* (olfactory marker protein) promoter. We analyzed the neuronal activity of olfactory system by blue light using an immediate early gene, *c-fos,* a marker for neuronal activity, as well as by using a genetically encoded calcium indicator, GCaMP in the transgenic zebrafish.

To examine whether the photoactivation of olfactory sensory neurons affect zebrafish behavior and to investigate which sensory modality, vision or olfaction, dominates decision-making, we designed a behavioral paradigm in which the innate phototaxis of zebrafish larvae and the presence of an aversive odorant are conflicted. We found that the wild type zebrafish avoided the arm with the odor stimulus despite that this arm was well illuminated. However, the transgenic zebrafish were insensitive to the aversive odorant when stimulated by the ChR2 specific blue light. These results suggest that olfaction is a critical sensory input in decision making in wild type larval zebrafish as it is able to override phototaxis, an olfaction driven response that is abolished in transgenic fish upon optogenetic activation of the olfactory sensory neurons with the ChR2 specific blue light. These results indicate that the transgenic fish will be useful to investigate the functional analysis of olfactory circuits or related behavior more easily. 

## 2. Results

### 2.1. Establishment and Characterization of Transgenic Zebrafish Expressing Channelrhodopsin-2 in Olfactory Sensory Neurons

**Figure 1:**
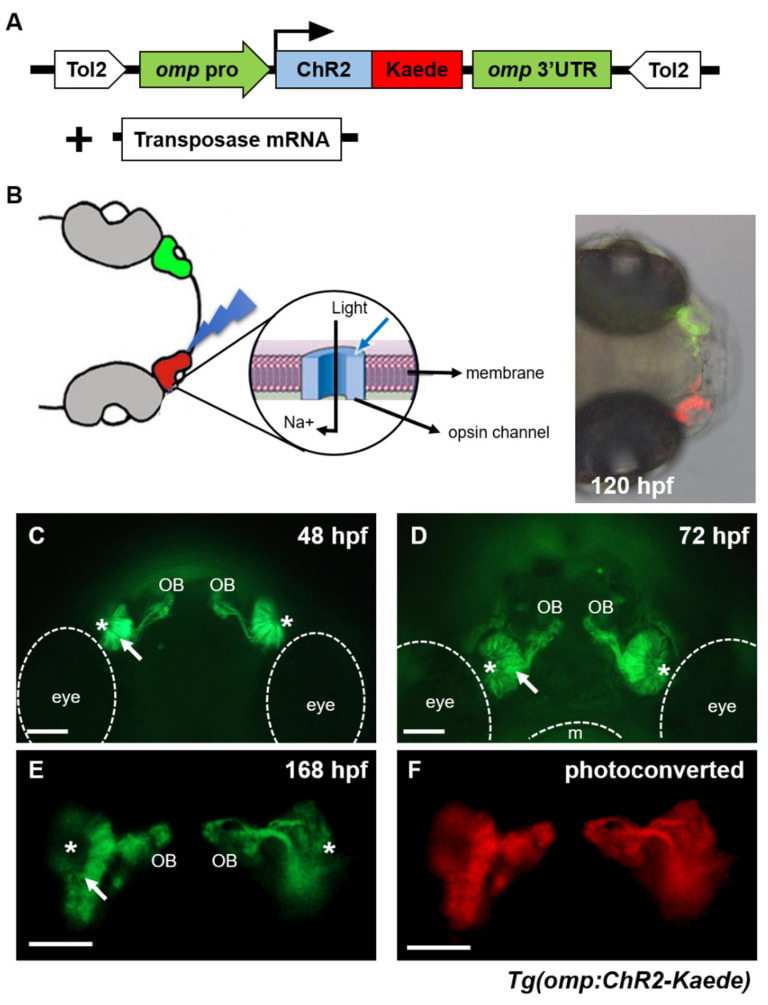
Generation of a transgenic zebrafish expressing ChR2-Kaede in olfactory sensory neurons. (**A**) Generation of transgenic fish using the expression construct of *omp*-driven ChR2-Kaede with transposase mRNA. (**B**) Schematic view of the transgenic zebrafish expressing channelrhodopsin-2 (ChR2) in olfactory sensory neurons. The green fluorescence of Kaede was photo-converted to red color, as an indicator for activation. (**C**,**D**) Ventral view of the head of the transgenic zebrafish embryos at 48 hpf and 72 hpf. (**E**,**F**) Dorsal view of the transgenic fish at 168 hpf. The Kaede protein of OSNs was photoconverted by UV light green to red fluorescence (**F**). Asterisks indicate the nasal pit, and arrows indicate the olfactory epithelium. m, mouth; OB, olfactory bulb. Scale bars, 50 μm.

Channelrhodopsins (ChR) transport various ions over the membrane in response to light and have been used to manipulate specified neuronal activity and behavior in various model animals [18,19,20]. We generated a transgenic zebrafish line carrying the *omp* promoter-driven ChR2-Kaede fusion gene to control neuronal activity of olfactory sensory neurons (OSNs) by blue light (Figure 1A,B). Olfactory marker protein, encoded by *omp* gene, is specifically expressed in the vertebrate olfactory sensory neurons [21]. We used the upstream promoter and 3′-untranslated region (3′-UTR) of the zebrafish *omp* gene [22] and newly generated a fusion protein of ChR2 with Kaede. The latter is a fluorescent protein and has property of photo-conversion from green to red by ultraviolet (UV) light [23,24]. Subsequently, we generated a transgenic zebrafish by microinjecting plasmid DNA and Tol2 transposase mRNA into fertilized eggs (Figure 1A, Figures S1 and S2). We wanted to activate one side of the same embryo. The other side of the embryo is used as an internal control. We fused Kaede to mark a region of light irradiation because the Kaede protein converted green to red upon light delivery as employed before [25].

The fluorescence of the ChR2-Kaede fusion protein in transgenic zebrafish fish first appeared in the olfactory epithelium at 48 h post fertilization (hpf). The olfactory sensory neurons projected their axons toward the developing brain, and their axon terminals were observed mainly around the developing olfactory bulb (OB) at 48 hpf (Figure 1C). At 72 hpf, the olfactory epithelium and nasal pit became expanded and olfactory nerve bundles were thickened (Figure 1D). ChR2-Keade expression in olfactory sensory neurons was observed by 168 hpf (Figure 1E) and continued to adulthood (data not shown). By UV photo-activation, the green fluorescence of Kaede was photo-converted to red color (Figure 1E,F). Kaede is used as reporter to visualize olfactory sensory neurons and to mark regions excited by light.

### 2.2. Evaluation of Light Induced Neuronal Activity Using Immediate Early Gene in the Transgenic Fish

**Figure 2:**
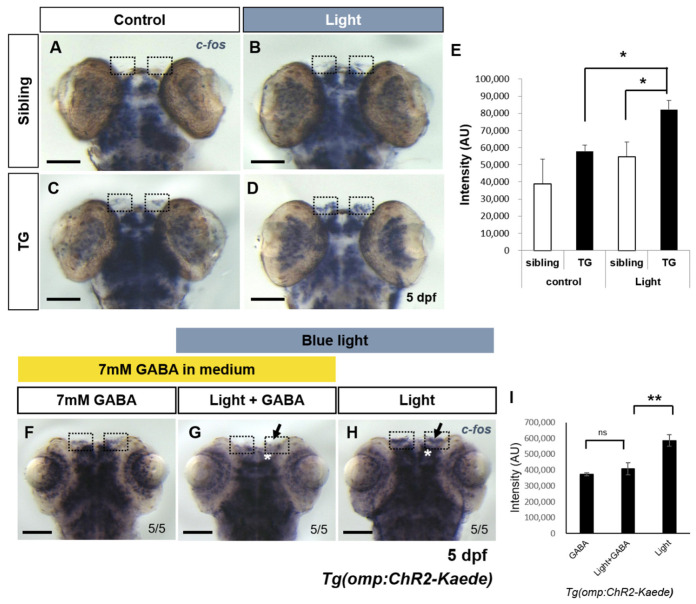
Expression analysis of *c-fos* for evaluating light-induced neuronal activity in transgenic fish. (**A**–**D**) Whole-mount in situ hybridization of *c-fos* in the transgenic fish and non-transgenic siblings at 5 dpf. (**A**) Control non-transgenic fish. (**B**) Light-irradiated non-transgenic fish. (**C**) Control transgenic fish. (**D**) Light-irradiated transgenic fish. Dotted boxes indicate olfactory epithelium of 5 dpf zebrafish. In light-irradiated transgenic fish, *c-fos* expression was ectopically induced at the olfactory epithelium (**D**). (**E**) Quantitative analysis of *c-fos* expression in olfactory epithelium. Intensity of *c-fos* transcripts in olfactory epithelium (dotted boxes) was measured by Image J. *n* = 4 for control sibling; *n* = 4 for control TG; *n* = 4 for light-irradiated sibling; and *n* = 5 for light-irradiated TG. (Tg light vs. Tg control, *p* = 0.0095; Tg light vs. sibling light, *p* = 0.04181). (**F**–**H**) Suppression of light-evoked odor response by GABA application in transgenic fish expressing ChR2 in the olfactory sensory neurons. (**F**) 7mM GABA treated control transgenic zebrafish at 5 dpf. (**G**) Optically stimulated transgenic zebrafish with GABA. (**H**) Light-irradiated transgenic zebrafish. Black arrow indicates olfactory epithelium and asterisk marked olfactory bulb. (**I**) Intensity of *c-fos* expression in olfactory epithelium upon GABA treatment. *n* = 5 for each experiment. (Tg light vs. Tg light + GABA, *p* = 0.005). All values are represented as mean ± S.E.M; * *p* < 0.05 and ** *p* < 0.005. *p* value was determined by unpaired t test. ns, not significant. Scale bars: 100 μm.

To evaluate ChR2 specific blue light-evoked neuronal activity of olfactory region in the newly generated transgenic fish, we quantified transcriptional activity of an immediate early gene, *c-fos.* Immediate early genes (IEGs) respond rapidly and transiently to a variety of cellular stimuli like neuronal depolarization [26,27,28]. Odor evoked neuronal activity using *c-fos* in rat was also reported [29,30]. In the absence of sensory stimulation, the brain generally expresses low basal levels of IEGs. We performed whole-mount in situ hybridization to analyze mRNA levels of *c-fos* after blue light irradiation at olfactory organ instead of an odor stimulus. ChR2 specific blue light was irradiated at the olfactory organ of 5 day old wild type and transgenic (TG) zebrafish larvae for 5 s. Thirty minutes after the irradiation the subjects were fixed. *c-fos* expression was ectopically induced in the olfactory epithelium of light-irradiated transgenic fish (Figure 2D). In contrast, blue light irradiated wild type and non-irradiated transgenic and wild type fish showed basal level of *c-fos* expression in the olfactory epithelium (Figure 2A–C). The intensity of *c-fos* transcript in the region of olfactory epithelium was measured using Image J software (Figure 2E). The intensity in the blue light-irradiated transgenic fish was higher than non-irradiated transgenic fish and light-irradiated wild-type fish. These results indi––cate that optical stimulation in the transgenic fish elicited neuronal activity in the olfactory epithelium. Since ChR2 responds to blue light of approximately 470 nm wavelength [15], we performed whole-mount in situ hybridization in the transgenic fish under various light conditions to confirm that the blue light-evoked *c-fos* expression in the transgenic fish was indeed due to ChR2. Under the blue (480 nm), UV (420 nm) and yellow (610 nm) light sources on a dissecting microscope, the transgenic zebrafish were irradiated and then fixed. In the siblings, expression of *c-fos* remained at basal level under these light conditions (Figure S3). Transgenic fish, however, irradiated with blue or UV light source showed induction of *c-fos* in olfactory epithelium and olfactory bulb. On the other hand, the yellow light irradiated transgenic fish displayed basal level of *c-fos* expression, similar to non-transgenic control fish (Figure S3). 

### 2.3. Inhibition of Light-Evoked Activity by GABA Agonist in Transgenic Fish 

GABA (gamma-aminobutyric acid) is the primary inhibitory neuro-transmitter and leads to a hyperpolarization of target neuron in most vertebrate brain areas. GABAergic interneurons in the olfactory bulb of vertebrates play crucial roles in the processing of odor-evoked patterns. In zebrafish, the application of GABA (5–10 mM) suppressed odor responses in the developing olfactory bulb [31,32]. To examine whether GABAergic activation suppresses the ChR2 specific blue light-evoked odor responses in the transgenic fish, we analyzed the expression level of *c-fos* after GABA administration in the transgenic zebrafish as described before [31]. In the ChR2 specific blue light-irradiated transgenic fish, transcripts of *c-fos* were detected at the olfactory epithelium (Figure 2H). In contrast, in the ChR2 specific blue light-irradiated transgenic fish that were administered 7 mM GABA for 1 h, the area where *c-fos* positive neurons were found was reduced in the olfactory bulb as well as in olfactory epithelium compared to control (Figure 2G,I). Only GABA treated (no ChR2 specific blue light) transgenic fish showed normal basal level of *c-fos* expression in the olfactory epithelium (Figure 2F). These results showed that GABA could inhibit the ChR2 specific blue light evoked odor responses in the transgenic fish. 

### 2.4. Light-Driven Neuronal Activity Analysis in the Olfactory Brain Area Using a Genetically Encoded Calcium Indicator

**Figure 3:**
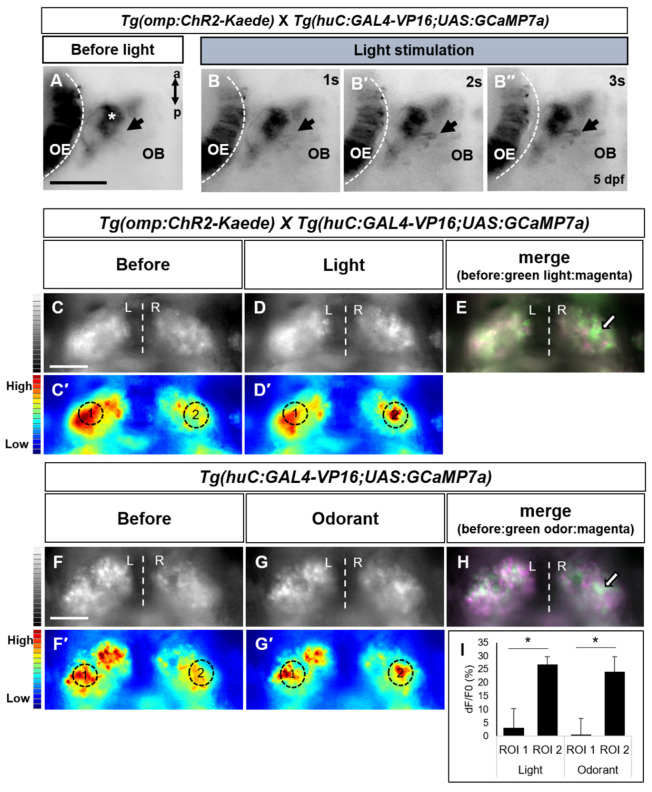
Analysis of calcium transient in olfactory bulb and habenula evoked light/odor stimuli. (**A**) Olfactory epithelium (OE) and olfactory bulb (OB) of transgenic fish before light irradiation. (**B**,**B′**,**B″**) After light stimulation, calcium signals were detected in olfactory bulb. Asterisk indicates axon terminals of olfactory sensory neurons and arrow indicates newly emerging cell bodies by optical stimulation. a, anterior; p posterior. Scale bar: 50 μm. (**C**–**H**) Odor responses of habenula in transgenic zebrafish lines. (**C**–**D′**) In *Tg(omp:ChR2-Kaede);Tg(huc:GAL4-VP16;uas:GCaMP7a)*, habenular neuron responses to light. (**F**–**G′**) Odorant evoked habenular response in *Tg(huc:GAL4-VP16;uas:GCaMP7a)*. Cells before stimulated were colored in magenta, and cells responding to light/odor were colored in green (**E**,**H**). Data are representative of at least two independent experiments. (**I**) Bar chart showing average dF/F0 of ROI 1 (left habenula) or ROI 2 (right habenula) in zebrafish brain expressing GCaMP7a upon the ChR2 driven light or odor activation (*n* = 3 each group). Dotted circles depict ROI of quantitative analysis. All values represent mean ± S.E.M. * *p* < 0.05. *p* value was determined by Student’s t test. L, left; R, right; ROI, region of interest. Scale bars: 50 μm.

Intracellular calcium transients reflect the electrical events in neurons, and calcium imaging with calcium indicators is routinely used in neuroscience to assess neuronal activity. To observe neuronal activity triggered by ChR2 specific blue light in central nervous system responsible for olfactory stimulus processing in the transgenic fish, we used a genetically encoded calcium indicator (GECI), GCaMP transgenic line. We generated triple transgenic fish *Tg(huc:gal4-VP16;uas:GCaMP7a;omp:ChR2-Kaede)* to obtain neuronal cell specific GCaMP expression [33,34]. Subsequently, we monitored neuronal activity induced by ChR2 specific blue light in brain areas responsible for olfactory stimulus processing, including the olfactory bulb, telencephalon and habenula in the transgenic fish carrying ChR2 (Figure 3). Before blue light activation, calcium signal was found absent in the olfactory bulb (Figure 3A). After blue light irradiation, we observed a GCaMP signal in some cell bodies beside axon terminals of olfactory sensory neurons. These signals were assumed to be in mitral cells in the glomerulus of the olfactory bulb. The signals increased with the course of time (Figure 3B,B′,B″). In addition, the intensity of green fluorescence of Kaede induced by light decreased in the olfactory epithelium showing activation of the ChR2-Kaede fusion protein. In the telencephalon, axon projections of mitral cells from olfactory bulb to higher-brain centers were observed (Figure S4). This pattern observed in the telencephalon of transgenic fish is similar to that published in a previous study on axon projections of mitral cells [35]. 

The habenula is a small, complex, and evolutionary conserved brain structure located at the dorsal end of the diencephalon. The habenula is composed of two parts based on connectivity and functional heterogeneity. The medial and lateral habenulae in mammals are homologous to the dorsal and ventral habenulae of fish, respectively. The innervation of mitral cell into the right habenula in zebrafish also has been reported [36,37,38,39]. We monitored neuronal activity of habenulae in the *Tg(omp:ChR2-Kaede)* crossed with *Tg(huc:GAL4;uas:GCaMP7a)* transgenic fish. First, strong and spontaneous activity of GCaMP was detected throughout the habenula compared to the other brain region in the transgenic fish (Figure 3C–G′). The spontaneous activity in these areas is believed to be associated with numerous behavioral functions, including sensory processing, learning and sleeping [40]. We observed calcium transients of the right side of habenula when light was irradiated (Figure 3D,D′) consistent with the innervation of mitral cells into the right habenula [39]. Left-Right (LR) asymmetries in habenular nuclei coupled with their asymmetric afferent input may underlie the functional asymmetries. Dreosti and colleagues suggested that most habenular neurons responding to odors are found in the right side of habenula, and disruption of this asymmetry may affect sensory processing [36]. To confirm whether the calcium response in the right habenula was elicited by light-driven odor response, we employed an odorant in the *Tg(huc:GAL4-VP16;uas:GCaMP7a)* fish. We examined whether the odor response could recapitulate the same calcium transient pattern in habenular neurons. As in the optically stimulated *Tg(omp:ChR2-Kaede)* fish, similar pattern of calcium transient that was showed in the right habenula was observed in the odorant treated *Tg(huc:GAL4-VP16;uas:GCaMP7a)* fish (Figure 3G, G′). Although the size and shape of the habenula was slightly different, the right side of habenula was activated in both light-illuminated transgenic fish and odorant treated fish. Moreover, the activated region of habenular neurons was located more dorsally. Through a more detailed comparison, we observed that the activated region of habenula was similar (Figure 3E,H and Figure S5). Additionally, we found that the dF/F0 at the right habenula region (ROI 2) was similar in ChR2-light activated transgenic fish and odor activated fish. Compared to this, the dF/F0 of the left habenula (ROI 1) was significantly lower than right habenula (Figure 3I). The activated region showed consistency with previous findings that showed olfactory stimulus induced responses in the right habenula [36,39,40]. The slight variability of habenula and positions of activated neurons was likely due to developmental variability across individual zebrafish. 

### 2.5. Behavioral Adaptation to Light Evoked Odor Response in the Transgenic Zebrafish

**Figure 4:**
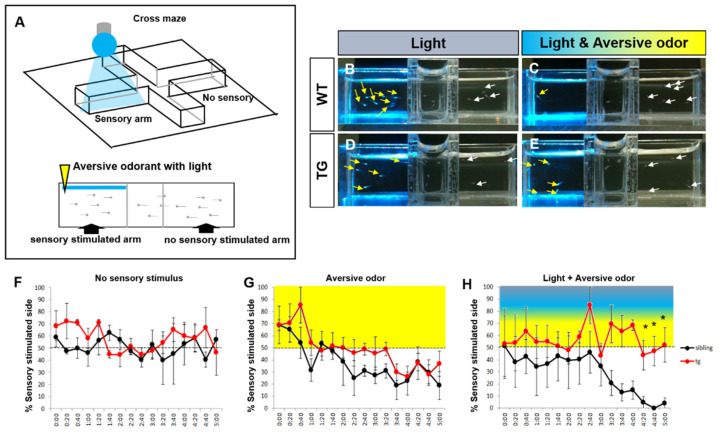
Light and odorant-driven behavior in transgenic zebrafish. (**A**) Schematic diagram of the conflicting sensory cues-based behavioral test. In a cross-shaped maze, blue light illumination and odorant treatment was performed. The light and the odor cue were both presented in the same (left) arm. (**B**–**E**) Images of zebrafish in the behavioral apparatus. The control sibling fish swam toward the blue light illuminated site (**B**), but when the light was co-presented with the aversive odor cue, they swam away from the stimulus presentation arm (**C**). The transgenic fish showed no avoidance behavior when the blue light and aversive odor were co-presented (**E**). Yellow arrows indicate the zebrafish larvae in the stimulus arm; white arrows depict the zebrafish in the no stimulus arm. (**F**–**H**) Behavioral response to blue light and aversive odor was measured by the percent of larvae counted in stimulus arm of the cross-shaped maze over time (20 fishes). (**F**) No sensory stimulus. (**G**) The aversive odor alone. (**H**) Blue light and aversive odor co-presented. Dotted lines indicate 50 % of the number of fish in the stimulus arm (chance level performance). All values represent mean ± S.E.M. * *p* < 0.05. *p* value was determined by Student’s *t* test. *n* = 3 clutches from three independent experiments.

Next, we analyzed the behavior of our transgenic fish to determine how photo-activation of OSNs affects behavioral aspects of zebrafish larvae. Sensory information is abundant in the environment of zebrafish. Distinct brain regions receive different sensory information and integrate it to allow appropriate behavioral responses. For example, olfactory stimulation is known to regulate processing of visual signals [41]. However, when animals receive conflicting sensory cues such as aversive odors and appetitive light cues, mechanisms underlying the animal’s behavioral responses are not fully understood. In previous experiments, we found an aroma oil that zebrafish larvae avoided in a cross-shaped maze. Additionally, zebrafish larvae showed phototaxis, swimming toward a light source, in light-dark choice task [8,9,10]. Combining these opposing sensory cues, we designed a behavioral experiment as shown in Figure 4. In this experiment, both wild type and transgenic zebrafish larvae swam toward the blue light-illuminated side (Figure 4B). When we administered an aversive odorant (*Citrus paradisi*) for 5 min, both wild type and transgenic fish swam away from the olfactory cue (Figure 4G). When we administered the odorant and blue light together, wild type fish swam away from the arm where these cues were presented (Figure 4C,H). This suggests that the wild type zebrafish avoid the aversive odorant despite the conflict due to their innate phototaxis. In contrast, the transgenic fish did not show preference for either the light or the odor cue (Figure 4E,H). Whether this alteration of behavior is due to processes similar to those underlying olfactory adaptation [42] is an empirical question that requires experimental analyses in the future. Adaptation to odors is known to reduce perception of an odor by repeated stimulation [42]. Taken together, these results suggest that optical stimulation of OSNs in transgenic zebrafish could alter the behavior of zebrafish larvae. In addition, we showed that an aversive olfactory cue can drive the behavior of zebrafish dominating over phototaxis, and this response can be altered by prolonged olfactory stimulation, likely due to olfactory adaptation. 

## 3. Discussion

Optogenetic methods allow activation/deactivation of specified neuronal subpopulations and the monitoring of neuronal activity in the intact brain, methods that help us understand how changes in the activity of specific neuronal circuits contribute to behavior. We employed these tools and generated a transgenic zebrafish model expressing the ChR2-Kaede fusion protein in the olfactory sensory neurons to understand the function of neural circuits involved in olfactory processing in zebrafish. The advantage of this optogenetic technique is the non-invasive application of photo-activation which allows specific stimulation of OSNs within intact neuronal networks. Because the larval zebrafish is transparent and displays a rich repertoire of behaviors, we could combine optogenetic methods to activate OSNs and behavioral analysis. In the invertebrate *Drosophila* model system, optogenetic approaches have been already well established to manipulate the activity of specific OSNs and odor-guided behaviors [43]. However, there are few studies in zebrafish olfactory research using optogenetic applications. In line with this, we successfully established a new transgenic zebrafish larval model allowing manipulation of the activity of OSNs by light. In the current study, we used *omp* promoter, which is expressed specifically in ciliated OSNs—one of several types of OSN; others include microvillous OSNs which are distinct from ciliated one in zebrafish [21]. Since we have activated only ciliated OSNs in this study, it remains to be investigated which odorant receptor contributes to olfactory behaviors by using a specific promoter of distinct olfactory receptor. 

Immediate early genes (IEGs) respond rapidly and transiently to a variety of cellular stimuli [26,44]. Brain mapping using *c-fos* mRNA has been often employed in neuroscience research [8,45]. Here, we analyzed light-induced neuronal activity in olfactory epithelium by quantifying the expression of an immediate early gene, *c-fos,* demonstrating that our system effectively activates OSNs. The blue light-evoked neuronal activity in transgenic fish was repressed in the olfactory epithelium and olfactory bulb by administration of the inhibitory neurotransmitter GABA. These results suggest that neuronal activity by light stimulation was regulated by the GABAergic system which influences odor-evoked activities [31,32]. 

To monitor activity of central brain areas involved in olfactory stimulus processing more precisely, we used a genetically encoded calcium indicator, GCaMP. We observed calcium transients in the mitral cell region of the olfactory bulb and in the olfactory tract of telencephalon region. Additionally, the right habenular neurons responded to blue light in the transgenic fish carrying ChR2. When an odorant was administered in the *Tg(huc:GAL4-VP16;uas:GCaMP7a)*, similar neuron population in the habenula showed calcium signals by GCaMP. The activated neuronal population was observed in the right dorsal habenula. These findings were consistent with recently reported results about the zebrafish habenula: olfactory mitral cell axons innervate into the right habenula and the dorsal habenula is known to receive various sensory inputs including olfactory stimuli [35,36,39]. The habenula has an asymmetric structure and is divided into medial/dorsal and lateral/ventral sections both, in mammals and in fish. The left-right differences of this bilateral brain structure are associated with distinct neuronal populations belonging to specific neurotransmitter systems leading to functionally distinct roles the left vs the right habenula plays [37,38,39,40]. When the asymmetry was disrupted, the sensory processing function of the habenula was found to be impaired [30,36]. Our results on the blue light-evoked olfactory responses in the transgenic fish through GCaMP in right and dorsal habenular nuclei are consistent with the above-discussed previous findings. However, these analyses of the habenulae could not distinguish whether the responses were aversive or appetitive because the categorization of olfactory information such as attractive vs aversive olfactory cues already starts in the olfactory bulb [40]. Our *c-fos* and GCaMP analyses suggest that the blue light irradiation to transgenic fish could evoke neuronal activity in the olfactory epithelium and olfactory related brain regions including mitral cells, habenula. 

We next asked whether olfactory stimuli by light influences the behavior of zebrafish larvae. In our behavioral analysis, we utilized the known fact that zebrafish larvae exhibit phototaxis. We wanted to contrast this appetitive cue (light) with aversive olfactory cue to examine how manipulation of the olfactory system using optogenetic methods may alter stimulus preference, that is, decision making to go towards or away from a particular stimulus. The optogenetic method allowed us to use light both as an olfactory system controlling tool and also as a stimulus processed by the visual system. This behavioral analysis provides insights on how animals choose when conflicting sensory cues are present. In addition, the observation showing the aversive olfactory stimulus to be the most salient cue for decision making in zebrafish suggests that aversive cues are prominent when sensory modalities conflict.

Our combinatory analyses of photoactivation and odorant application in the transgenic and wild type zebrafish emphasize the importance of olfaction. Photoactivated transgenic fish showed less sensitivity compared to wild type, as desensitization occurs naturally upon prolonged exposure to the same olfactory cue [42,46]. One possible explanation for this result may be the cross talk between systems processing stimuli of different modalities in the brain. For example, olfactory stimuli can alter the processing of visual signals in the retina via neuronal projections linking the olfactory bulb to the retina, known as the olfactory-retinal circuit (ORC). Additionally, olfactory stimuli have been found to alter visual processing via regulation of concentration of dopamine in the retina [41,47]. 

The olfactory marker protein (*omp*) promoter was used to drive widespread expression of ChR2-Kaede in ciliated olfactory sensory neurons of zebrafish in this study. The optical-activation of the wide-ranging expression of ChR2-Kaede in olfactory sensory neurons could elicit the olfactory driven neuronal activity in olfactory epithelium, olfactory bulb and habenula. Nevertheless, whether the olfactory response was attractive or aversive could not be determined in the transgenic zebrafish. Because recognition and discrimination of odor depends upon the pattern of activation of the odorant receptors in OSNs [48], the global activation of OSNs by light in the transgenic fish is expected to be difficult in terms of motivational value in behavioral assays. 

In conclusion, we established a transgenic model to manipulate and monitor the effects of olfactory stimuli in living zebrafish. Analysis of the expression of an immediate early gene and using a transgenic approach for a calcium indicator, GCaMP, allowed us to evaluate neuronal activity changes induced by light in the olfactory stimulus processing system of the transgenic fish and also helped us study correlated behavioral responses. Taken together, our results suggest that the transgenic system employed here will be appropriate to study the neural circuit of olfactory behavior of zebrafish. Given the translational relevance of the zebrafish, our pioneering work may lead to better understanding of the neurobiological mechanisms of olfactory stimulus processing in other vertebrates as well.

## 4. Materials and Methods

### 4.1. Animals

Wild-type zebrafish (*Danio rerio*), outbred in our fish facility, were maintained at 28.5 °C in a 14/10 h light and dark cycle. The Animal Ethics Committee of Chungnam National University (CNU 00191) approved all animal experiments. Embryos were raised in embryo medium at 28.5 °C and embryonic stages were determined by standard method [49]. To inhibit melanization of embryos, phenylthiourea (1-phenyl 2-thiourea, PTU, Sigma, St. Louis, MI, USA) was administered at 10 hpf (hours post fertilization). Zebrafish were obtained from the Zebrafish Center for Disease Modeling.

### 4.2. Establishment Transgenic Zebrafish for the Omp-Driven ChR2-Kaede

#### 4.2.1. Construction of Expression Vector

To express ChR2 in zebrafish olfactory sensory neurons, firstly we isolated 5′ upstream promoter region and 3′ untranslated region (UTR) of the zebrafish olfactory marker protein, *omp* (AB073551) from the Pomp-GFP expression vector (a gift from Tomoyuki Yoshida, University of Tokyo, Tokyo, Japan) [22]. The 2.7 kb of promoter region and the 3 kb of 3′-untranslated (UTR) region were amplified by PCR following primers. *omp* promoter forward primer5′-GCG TCG ACT GTT TTG GTG GCA GAT CTC G-3′ *omp* promoter reverse primer 5′-CGG ATC CTG CGT CTA CTG TGT GTC CTT GG-3′; *omp* 3′UTR forward primer 5′-TGG GAA TTC GAG AAA ATG AAG GGT ACA ACG G-3′ omp 3′UTR (reverse) 5′-GTC TAG ATG TCA GAA GTG CTT GCT AAG GCT-3′. The primers were designed by using zebrafish genome database (http://www.ensembl.org, ensemble ID: ENSDARG00000032380) and containing some restriction enzyme recognition sequences and the PCR products were subcloned into expression vector, mini-Tol2 EGFP. To express ChR2 and Kaede fused protein in zebrafish olfactory sensory neurons, the primers were designed to insert specific restriction enzyme recognition (cutting) sites for Kaede (AB085641) [23,24], amplified by PCR and subcloned in-frame into C-terminus of ChR2 (AF461397) in pPD96.52 vector using *Kpn I* and *EcoR I* (Takara Bio Inc., Shiga, Japan) restriction enzymes [18,50]. pPD96.52_ChR2-YFP vector was provided by Prof. Junho Lee [50]. Kaede forward primer (5′-TCG GCC AGG TAC CGA TGA GTC TGA T-3′) containing *Kpn I* and reverse primer (5′-TTG AAT CCC TAC TTG ACG TTG TCC GGC A-3′) containing *EcoR I*. The fusion nucleotide was cut with *BamH I* and *EcoR I* subcloned into mini-Tol2 expression vector containing *omp* promoter and 3′UTR.

#### 4.2.2. Microinjection and Generation of Transgenic Zebrafish Lines

Zebrafish embryos were injected with Tol2-based expression construct and Tol2 transposase mRNA at 1-cell stage. To generate stable transgenic line, injected embryos were raised to adulthood. The injected fish were crossed with wild type fish and the offspring were screened for Kaede expression using fluorescence microscopy (Leica MZ16FA, Wetzlar, Germany).

### 4.3. Whole-Mount In Situ Hybridization

Immediate early gene, *c-fos* (NM_205569) was amplified by RT-PCR using the following primers (5′-GGG ATC CGA CAG GAT GAT GTT TAC CAG CCT T-3′ and 5′-CCT CGA GGT GGA ATC TCA AAG AGT GAG GAG G-3′). The PCR product was cloned into the pGEM-T-easy vector (Promega, Madison, WI, USA). To synthesize RNA probe, pGEM-T-easy vector containing *c-fos* was linearized with *Spe I* restriction enzyme. The linearized fragment was transcribed in vitro using T7 RNA polymerase (Thermo scientific, Waltham, MA, USA) and digoxigenin-labeled UTP (Roche, Basel, Switzerland). Whole-mount in situ hybridization was performed as previously described [51] with the anti-sense probe. To examine *c-fos* expression, embryos were fixed 30 min after 5 s-light irradiation with maximal intensity of the mercury lamp (DM 5000B, Leica, Wetzlar, Germany).

### 4.4. Light Stimulation and Calcium Imaging

To visualize calcium activity of light-driven olfactory stimulation in this transgenic system, we used transgenic zebrafish *Tg(huc:GAL4-VP16)* [33] and *Tg (uas:GCaMP7a)* [34]. These transgenic lines were obtained from the Koichi Kawakami lab through the central facility of NBRP Japan (RIKEN). The *Tg(omp:ChR2-Kaede)* fish were crossed with *Tg(huc:GAL4;uas:GCaMP7a)*. The triple transgenic zebrafish of 4-6 dpf were embedded in 5% methylcellulose (Sigma Chemical Co., St. Louis, MO, USA, M0512) in embryo medium (sea salt 0.006% Sigma, S9883). Each embryo was irradiated using a Leica DM 5000B upright compound microscope and 20×/0.5 NA objective equipped with a mercury lamp using a GFP filter (480nm). To target olfactory epithelium region, diaphragm was used. Odorant *Citrus paradisi* aroma oil was used in this experiment to monitor habenular activity. Calcium activity was observed using a fluorescence microscope (DM 5000B, Leica, Wetzlar, Germany) equipped with a CCD camera (DC300 FX, Leica, Wetzlar, Germany). Due to spectral overlap and subsequent cross-talk, the use of ChR2 specific blue light and GCaMP-aided imaging represents a problem. To minimize ChR2 activation during calcium imaging, we irradiated the olfactory epithelium first, and subsequently we adjusted the position of larvae to avoid direct olfactory region irradiation, which allowed us to focus our imaging on the other brain regions. To calculate dF/F0 we first measured the average intensity of GCaMP7a in the ROI 1 (left habenula) and ROI 2 (right habenula), before ChR2-light or odor activation as baseline using ImageJ (National Institute of Health, Bethesda, Maryland, MD, USA). After activation, we measured the average intensity and then we calculated dF/F0 by dividing intensity change (activation-before) by baseline intensity.

### 4.5. Behavioral Assay Odorant Treatment

In a cross-shaped maze (width 12.5 cm × height 8.5 cm, maze width 1 cm), 20 zebrafish larvae 5 dpf of age were placed. Then *Citrus paradisi* aroma oil was treated with DMSO (dimethyl sulfoxide, Junsei Chemical Co.,Ltd., Tokyo, Japan) with 1:1 volume ratio. The left arm (stimulus arm) of the cross maze with freely moving zebrafish larvae were irradiated using fluorescence microscope (Leica MZ16FA, Wetzlar, Germany) equipped with a mercury lamp using a GFP filter (480 nm). 2 uL of odorant (*Citrus paradisi* aroma oil) was slowly injected into the end of the stimulus arm. Upon stimulus presentation, the behavior of zebrafish larvae was recorded from the side view using a video camera (Sony, HDR-CX190, Tokyo, Japan). During 5-min-long behavioral test sessions, the number of fish in each arm (stimulus, S and no stimulus, N) was counted with 20 s interval.

## Data Availability

The data presented in this study are available on request from the corresponding author.

## Supplementary Materials

The following are available online at https://www.mdpi.com/article/10.3390/ijms22137191/s1.

## Abbreviations

ChR2	Channelrhodpsin
*omp*	olfactory marker protein
GABA	gamma-Aminobutyric acid
GECI	genetically encoded calcium indicator
OSNs	olfactory sensory neurons
