# Supplementary material for: Optogenetic Manipulation of Olfactory Responses in Transgenic Zebrafish: A Neurobiological and Behavioral Study
Source: Int J Mol Sci. 2021 Jul 3;22(13):7191. doi: 10.3390/ijms22137191 (PMC8269104; doi:10.3390/ijms22137191)
Supplement: Supplementary file 1 [file ijms-22-07191-s001.zip › ijms-1275380-supplementary.pdf]

Supplementary file

Optogenetic manipulation of olfactory responses in transgenic zebrafish: A neurobiological and behavioral study

Yun-Mi Jeong[1,2], Tae-Ik Choi[1], Kyu-Seok Hwang[1], Jeong-Soo Lee[2], Robert Gerlai[3*], and Cheol-Hee Kim[1*]

[a] Department of Biology, Chungnam National University, Daejeon 34134, South Korea

[d] Disease Target Structure Research Center, Korean Research Institute of Biosciences and Biotechnology, Daejeon 34141, Republic of Korea.

[c] Department of Psychology, University of Toronto Mississauga, Mississauga, ON L5L 1C6, Canada

[*]To whom correspondence should be addressed:

Email: robert.gerlai@utoronto.ca and zebrakim@cnu.ac.kr

Supplementary Figure

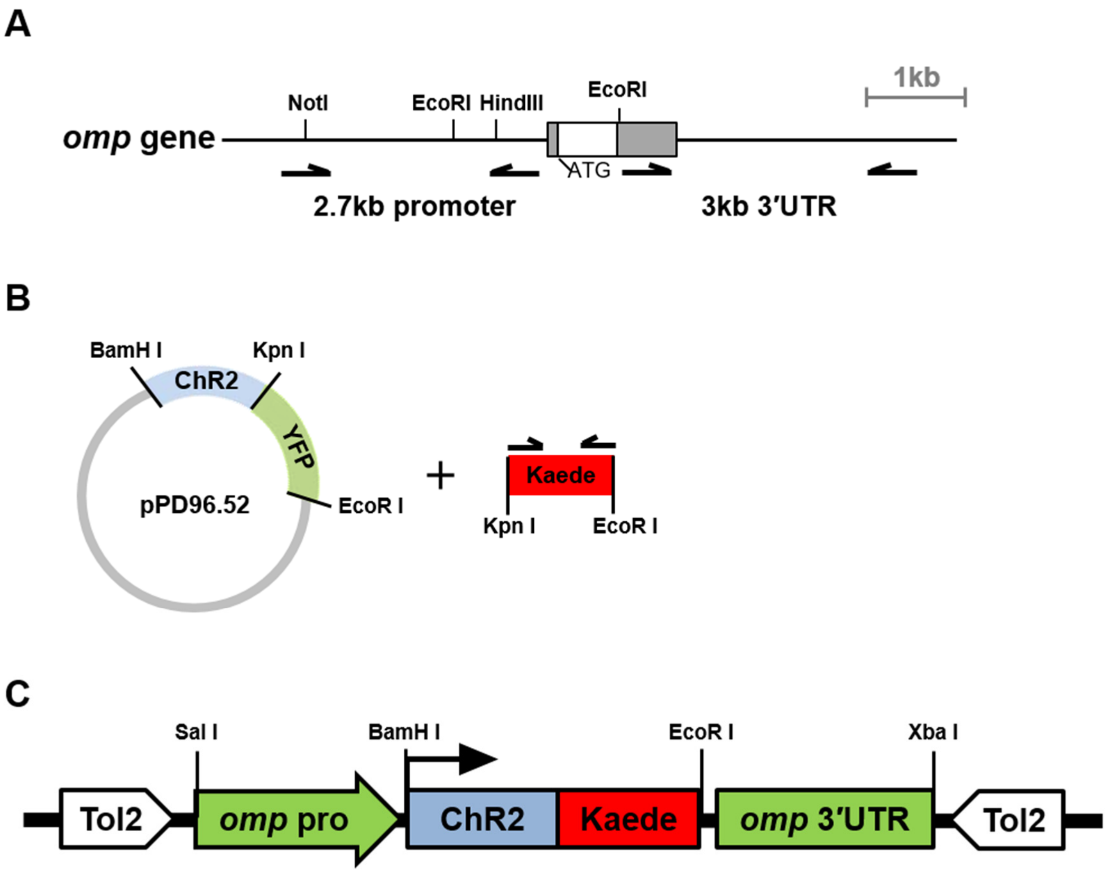

Figure S1. DNA contstructs for generating transgenic zebrafish lines expressing Channelrhodpsin-2/Kaede under the control of the *omp*. (A) Genomic structure and restriction enzyme sites of zebrafish *omp* gene. The 2.7kb 5′ upstream and 3kb downstream region of the *omp* translation initiation site was amplified by PCR. Black half arrows indicate primers. (B) Construction of fusion protein between ChR2 and Kaede. (C) Expression vector construction of *omp*-driven ChR2-Kaede.

```
atggattatggaggcgccctgagtgccgttgggcgcgagctgctatttgtaacgaaccca
 M  D  Y  G  G  A  L  S  A  V  G  R  E  L  L  F  V  T  N  P
gtagtcgtcaatggctctgtacttgtgcctgaggaccagtgttactgcgcgggctggatt
 V  V  V  N  G  S  V  L  V  P  E  D  Q  C  Y  C  A  G  W  I
gagtcgcgtggcacaaacggtgcccaaacggcgtcgaacgtgctgcaatggcttgctgct
 E  S  R  G  T  N  G  A  Q  T  A  S  N  V  L  Q  W  L  A  A
ggcttctccatcctactgcttatgttttacgcctaccaaacatggaagtcaacctgcggc
 G  F  S  I  L  L  L  M  F  Y  A  Y  Q  T  W  K  S  T  C  G
tgggaggagatctatgtgtgcgctatcgagatggtcaaggtgattctcgagttcttcttc
 W  E  E  I  Y  V  C  A  I  E  M  V  K  V  I  L  E  F  F  F
gagtttaagaacccgtccatgctgtatctagccacaggccaccgcgtccagtggttgcgt
 E  F  K  N  P  S  M  L  Y  L  A  T  G  H  R  V  Q  W  L  R
tacgccgagtggcttctcacctgcccggtcattctcattcgcctgtcaaacctgacgggc
 Y  A  E  W  L  L  T  C  P  V  I  L  I  R  L  S  N  L  T  G
ttgtccaacgactacagcaggcgcaccatgggtctgcttgtgtctgatattggcacaatt
 L  S  N  D  Y  S  R  R  T  M  G  L  L  V  S  D  I  G  T  I
gtgtggggcgccacttccgccatggccaccggatacgtcaaggtcatcttcttctgcctg
 V  W  G  A  T  S  A  M  A  T  G  Y  V  K  V  I  F  F  C  L
ggtctgtgttatggtgctaacacgttctttcacgctgccaaggcctacatcgagggttac
 G  L  C  Y  G  A  N  T  F  F  H  A  A  K  A  Y  I  E  G  Y
cacaccgtgccgaagggccggtgtcgccaggtggtgactggcatggcttggctcttcttc
 H  T  V  P  K  G  R  C  R  Q  V  V  T  G  M  A  W  L  F  F
gtatcatggggtatgttccccatcctgttcatcctcggccccgagggcttcggcgtcctg
 V  S  W  G  M  F  P  I  L  F  I  L  G  P  E  G  F  G  V  L
agcgtgtacggctccaccgtcggccacaccatcattgacctgatgtcgaagaactgctgg
 S  V  Y  G  S  T  V  G  H  T  I  I  D  L  M  S  K  N  C  W
ggtctgctcggccactacctgcgcgtgctgatccacgagcatatcctcatccacggcgac
 G  L  L  G  H  Y  L  R  V  L  I  H  E  H  I  L  I  H  G  D
attcgcaagaccaccaaattgaacattggtggcactgagattgaggtcgagacgctggtg
 I  R  K  T  T  K  L  N  I  G  G  T  E  I  E  V  E  T  L  V
gaggacgaggccgaggctggcgcggtaccgatgagtctgattaaaccagaaatgaagatc
 E  D  E  A  E  A  G  A  V  P  M  S  L  I  K  P  E  M  K  I
aagctgcttatggaaggcaatgtaaacgggcaccagtttgttattgagggagatggaaaa
 K  L  L  M  E  G  N  V  N  G  H  Q  F  V  I  E  G  D  G  K
ggccatccttttgagggaaaacagagtatggaccttgtagtcaaagaaggcgcacctctc
 G  H  P  F  E  G  K  Q  S  M  D  L  V  V  K  E  G  A  P  L
ccttttgcctacgatatcttgacaacagcattccattatggtaacagggtttttgctaaa
 P  F  A  Y  D  I  L  T  T  A  F  H  Y  G  N  R  V  F  A  K
tacccagaccatataccagactacttcaagcagtcgtttcccaaagggtttcttgggag
 Y  P  D  H  I  P  D  Y  F  K  Q  S  F  P  K  G  F  S  W  E
cgaagcctgatgttcgaggacgggggcgtttgcatcgctacaaatgacataacactgaaa
 R  S  L  M  F  E  D  G  G  V  C  I  A  T  N  D  I  T  L  K
ggagacactttttttaacaaagttcgatttgatggcgtaaactttccccaaatggtcct
 G  D  T  F  F  N  K  V  R  F  D  G  V  N  F  P  P  N  G  P
gttatgcagaagaagactctgaaatgggaggcatccactgagaaaatgtatttgcgtgat
 V  M  Q  K  K  T  L  K  W  E  A  S  T  E  K  M  Y  L  R  D
ggagtgttgacgggcgatattaccatggctctgctgcttaaaggagatgtccattaccga
 G  V  L  T  G  D  I  T  M  A  L  L  L  K  G  D  V  H  Y  R
tgtgacttcagaactacttacaaatctaggcaggagggtgtcaagttgccaggatatcac
 C  D  F  R  T  T  Y  K  S  R  Q  E  G  V  K  L  P  G  Y  H
tttgtcgatcactgcatcagcatattgaggcatgacaaagactacaacgaggttaagctg
 F  V  D  H  C  I  S  I  L  R  H  D  K  D  Y  N  E  V  K  L
tatgagcatgctgttgcccattctggattgccggacaacgtcaagtaa
 Y  E  H  A  V  A  H  S  G  L  P  D  N  V  K  -

ChR2-
Kaede
KpnI enzyme site
```

Figure S2. The fused nucleotides and amino acids sequences of Channelrhodopsin-2 (ChR2) and Kaede. Sequences of nucleotides and amino acids for fused ChR2 and Kaede. Box colored blue indicates DNA sequence of ChR2 and red color depicted is Kaede. *Kpn* I site is depicted by white box.

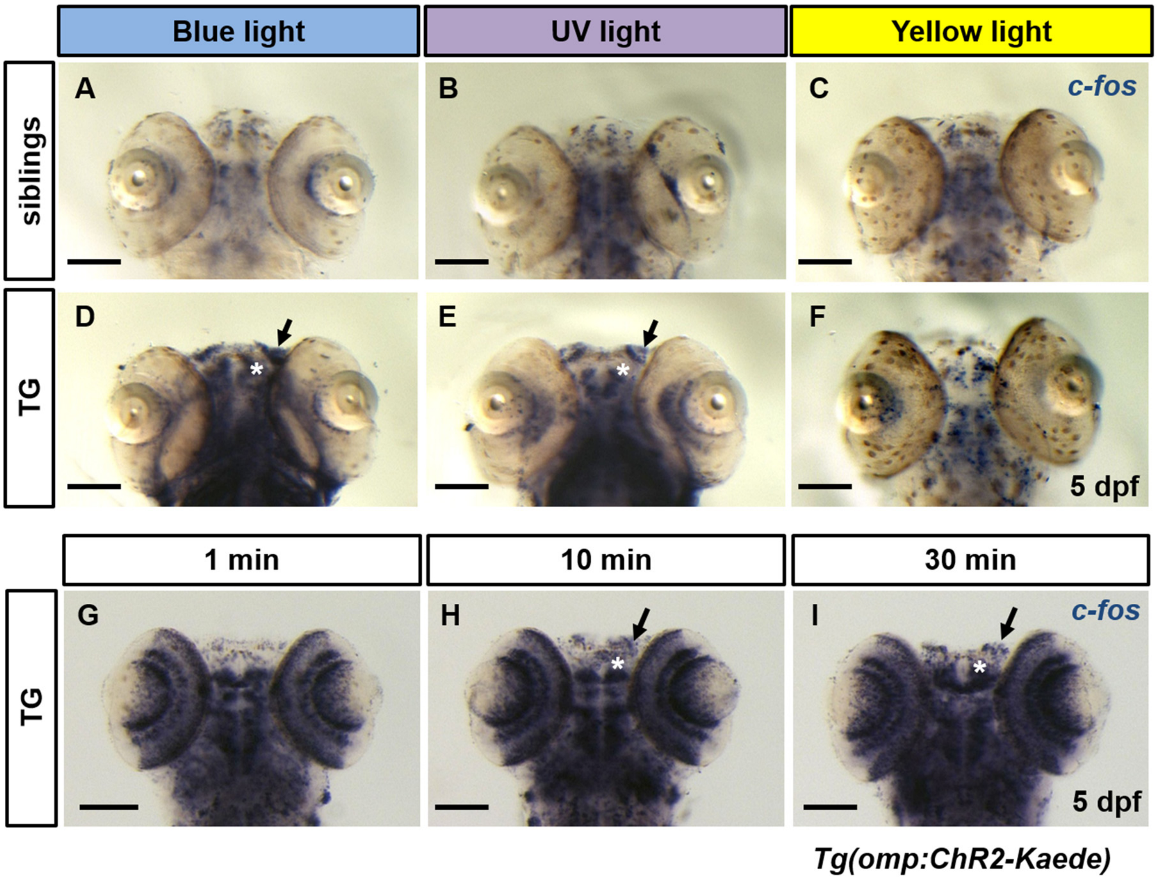

Figure S3. Assessment of light-induced neuronal activity in transgenic fish using immediate early gene, *c-fos*. (A-F) Images of *c-fos* expression according to various wavelength of light. (A-C) Sibling non-transgenic controls stimulated by each light source. (D-F) Transgenic fish irradiated by different light sources. In the blue light source and UV light source, the *c-fos* induction was observed (D, E). (G-I) Time-dependent *c-fos* induction after light irradiation in olfactory epithelium in the transgenic fish. Embryos were fixed at one minute (G), ten minutes (H), and thirty minutes (I) after blue light irradiation. Arrow and asterisk indicate olfactory epithelium and olfactory bulb, respectively. Scale bar: 100 µm.

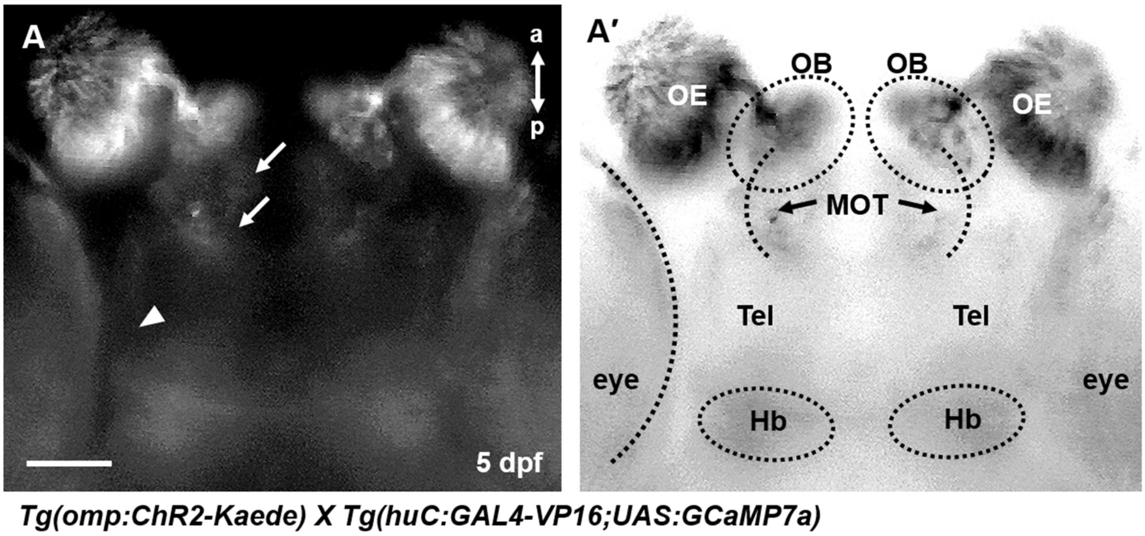

Figure S4. Calcium transient in the telencephalon and olfactory tract. (A) Calcium imaging of telencephalon and olfactory tract in the transgenic fish. White arrows indicate medial olfactory tract and arrowhead depict axon of mitral cell in the telencephalon area. Hb, habenula; OB, olfactory bulb; MOT, medial olfactory bulb. Scale bar: 50 μm

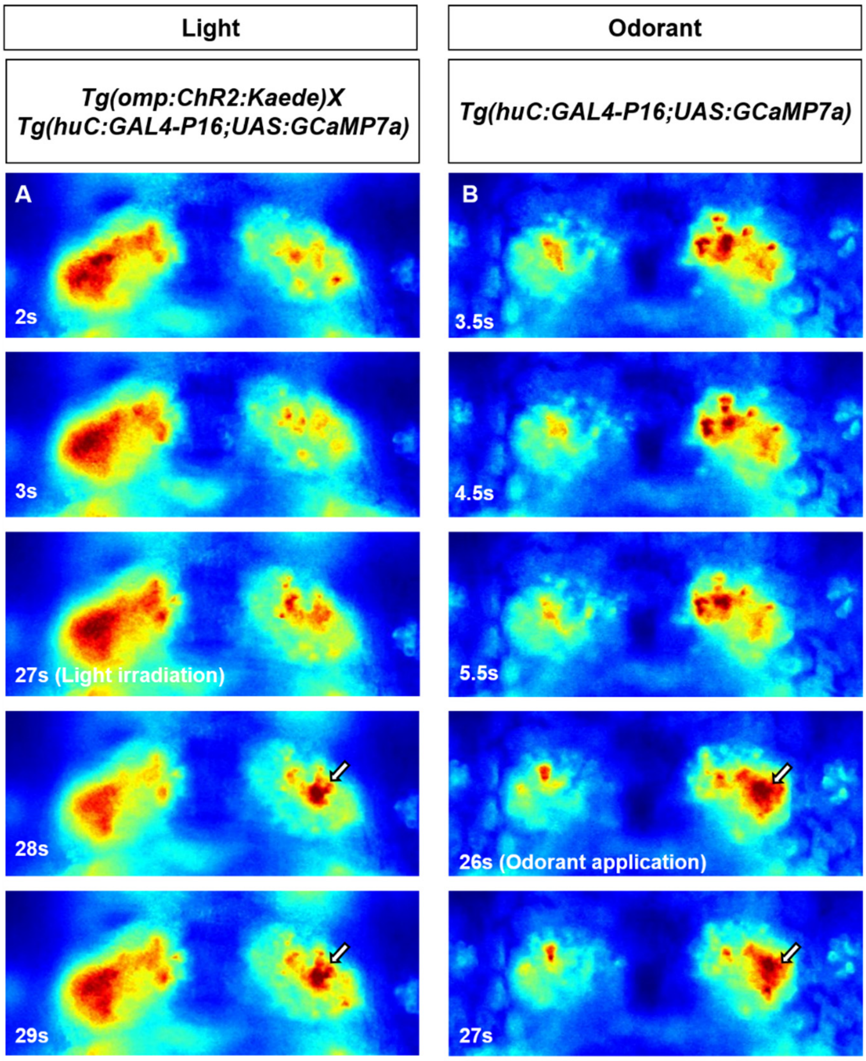

Figure S5. Habenular calcium responses by light-driven olfactory activation or odorant application. (A) Still images of calcium transient in the zebrafish habenula region. (A) Blue light-driven habenular responses in the *Tg(omp:ChR2-Kaede; huc:GAL4- VP16; UAS:GCaMP7a)* larvae (B) Habenular calcium response upon odorant (aroma oil, *Citrus paradisi*) treatment in the tg(*huc:GAL4- VP16; UAS:GCaMP7a)*. Arrows indicate increased calcium signal region of right habenula upon olfactory activation.
